# Durable complete response to pembrolizumab after BRAF/MEK inhibition in recurrent MSI-H/dMMR, BRAF V600E–mutant colon cancer: a case report

**DOI:** 10.3389/fonc.2026.1764072

**Published:** 2026-04-20

**Authors:** Wenyan Yu, Kaichun Li

**Affiliations:** Department of Oncology, Shanghai Fourth People’s Hospital, Tongji University School of Medicine, Shanghai, China

**Keywords:** BRAF V600E mutation, case report, colorectal cancer, immunotherapy, microsatellite instability-high, targeted therapy

## Abstract

Patients with metastatic colorectal cancer (mCRC) harboring both microsatellite instability–high/deficient mismatch repair (MSI-H/dMMR) and the BRAF V600E mutation represent a distinct clinicomolecular subgroup characterized by aggressive biology and distinct therapeutic challenges. While MSI-H/dMMR predicts sensitivity to immune checkpoint inhibitors (ICIs), the concurrent BRAF V600E mutation may contribute to secondary resistance or shortened durability of response in a subset of patients treated with ICI monotherapy. Here, we present the case of an 85-year-old female with locally advanced ascending colon cancer who experienced rapid recurrence and metastasis only four months after radical surgery. Molecular profiling confirmed MSI-H, dMMR, and BRAF V600E mutation. Given her advanced age and frailty, she was treated with a first-line combination of pembrolizumab, dabrafenib, and trametinib. Although the targeted therapy (dabrafenib/trametinib) was discontinued after approximately two months due to recurrent high-grade pyrexia, the patient continued on pembrolizumab maintenance. She achieved a radiographic complete response (CR) and has remained progression-free for over 26 months. This case highlights the potential synergy between MAPK pathway inhibition and immunotherapy, suggesting that even short-course targeted therapy may favorably remodel the tumor microenvironment to enable durable disease control in high-risk MSI-H/BRAF-mutant mCRC.

## Introduction

Microsatellite instability–high (MSI−H) status, resulting from deficient mismatch repair (dMMR), is a critical biomarker in colorectal cancer (CRC), found in approximately 5% of metastatic cases (1). These tumors are characterized by a high tumor mutational burden and intense lymphocytic infiltration, features that predict robust responsiveness to immune checkpoint inhibitors (ICIs) such as pembrolizumab (2, 3). Conversely, the BRAF V600E mutation, present in 8–10% of metastatic CRC (mCRC) patients, constitutively activates the MAPK pathway, driving cell proliferation and fostering an immunosuppressive microenvironment (4, 5).

The co-occurrence of MSI−H/dMMR and BRAF V600E creates a complex biological landscape. While these patients generally have better outcomes than those with BRAF−mutant microsatellite stable (MSS) tumors, they may still face a poorer prognosis compared to BRAF−wild−type MSI−H patients (6). Recent real−world data indicate that in treatment−naïve patients receiving first−line ICI monotherapy, the presence of a BRAF mutation is associated with a higher rate of secondary resistance (progression after 6 months), suggesting that ICI monotherapy alone may be insufficient for long−term control in a subset of this population (7).

Preclinical and translational studies suggest that inhibiting the MAPK pathway can modulate the tumor immune microenvironment, potentially sensitizing tumors to PD-1 blockade (8). This rationale underpins ongoing trials like SEAMARK, which evaluates the combination of pembrolizumab with BRAF/EGFR inhibitors (9). In this report, we describe an elderly patient with recurrent MSI-H/BRAF V600E mCRC who achieved a sustained complete response following a strategy of upfront combination therapy followed by de-escalation to ICI maintenance due to toxicity.

## Case presentation

**Figure 1:**
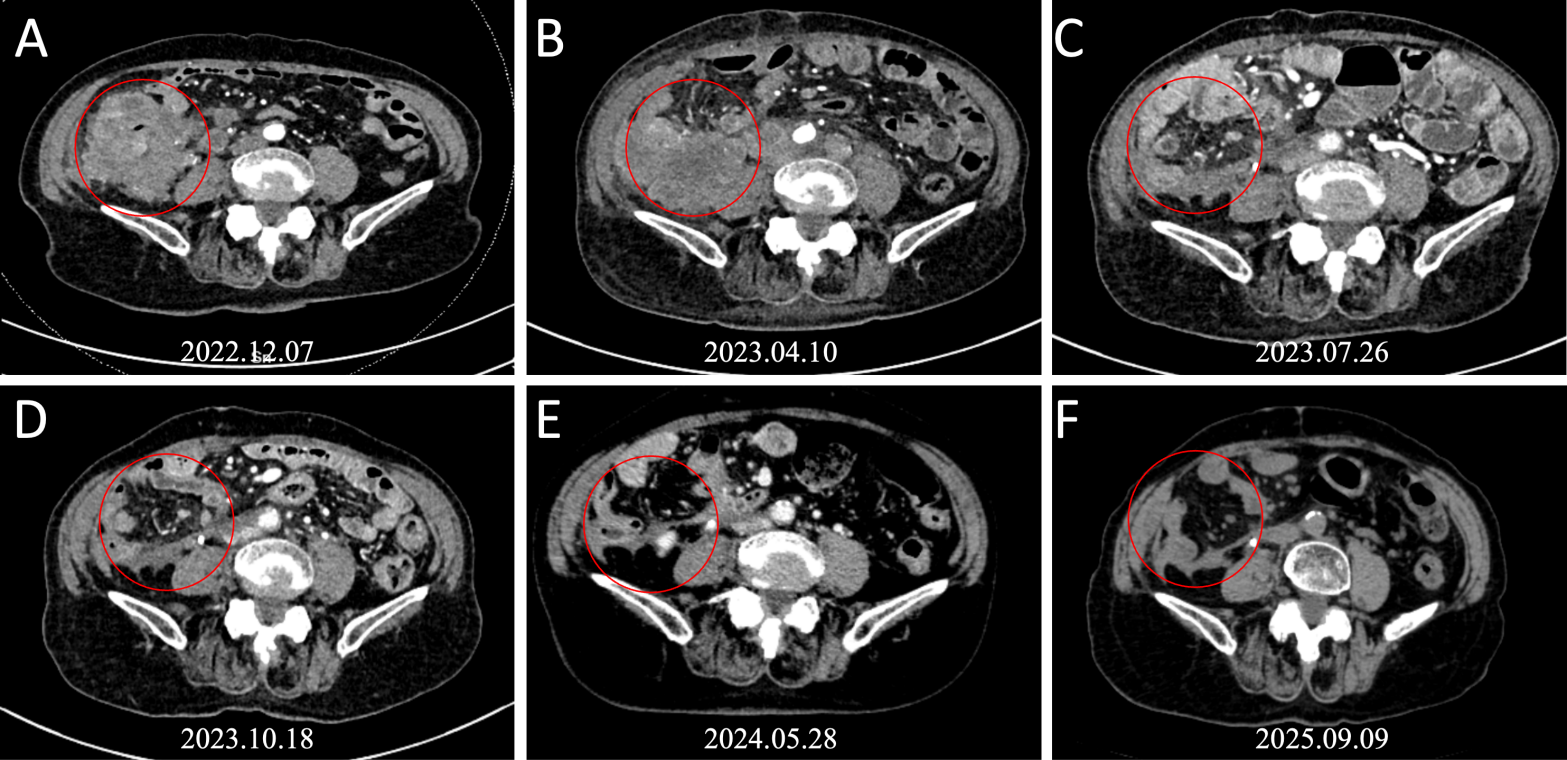
Serial contrast-enhanced abdominal CT scans. **(A)** Preoperative scan (December 7, 2022) showing thickening of the cecum and ascending colon. **(B)** Baseline scan at recurrence (April 10, 2023) prior to systemic therapy, showing a right lower quadrant mass and retroperitoneal lymphadenopathy. **(C)** Follow-up scan (July 26, 2023) after 3 months of combination therapy (pembrolizumab + dabrafenib + trametinib), showing partial response (PR). **(D, E)** Follow-up scans (May 2024 and others) during pembrolizumab maintenance, showing sustained tumor regression. **(F)** Most recent follow-up (September 9, 2025) confirming radiographic complete response (CR).

**Figure 2:**
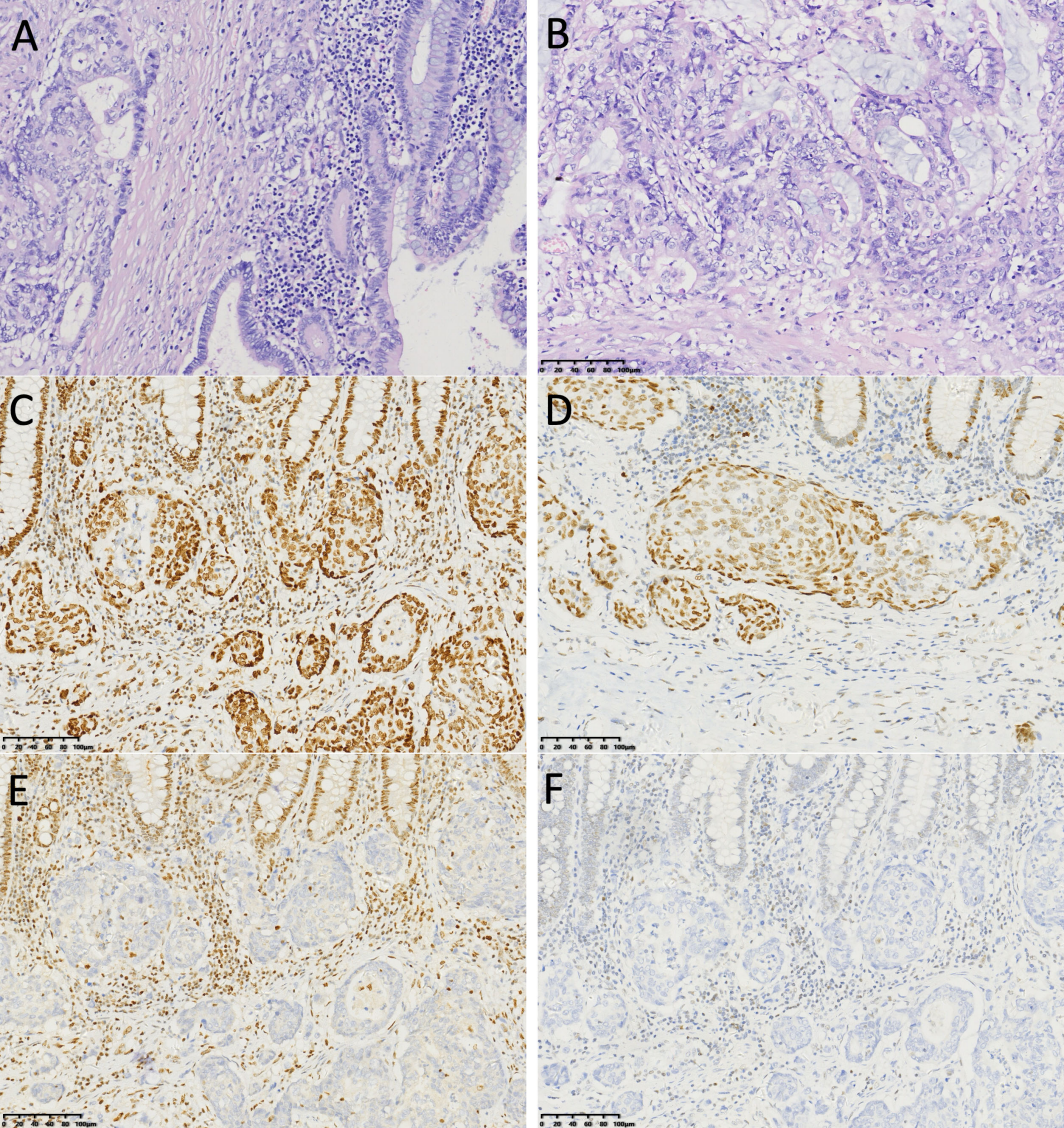
Histopathological and immunohistochemical features of the primary tumor. **(A, B)** HE staining at low magnification showing the overall architecture of the poorly differentiated adenocarcinoma. The images highlight extensive necrosis and a marked infiltrative growth pattern. IHC analysis of MMR proteins shows retained nuclear expression of **(C)** MSH2 and **(D)** MSH6, but loss of expression of **(E)** MLH1 and **(F)** PMS2. Scale bar = 100 μm applies to all panels.

An 85-year-old patient was admitted to the hospital with discomfort in the right abdomen. Laboratory tests showed: Hb 9.7 g/dL, CEA 3.35 ng/mL (normal value < 4.5 ng/mL), CA72-4 58.1 U/mL (normal value < 12 U/mL), CA125–60 U/mL (normal value < 35 U/mL). Computed tomography (CT) scan of the thorax to the abdomen showed thickening of the cecum and ascending colon wall (**Figure 1A**). The patient underwent laparoscopic radical colon cancer resection for colon malignancy on December 8, 2022. Postoperative pathology revealed a poorly differentiated adenocarcinoma with necrosis and a focal mucinous component (approximately 20%). Hematoxylin and Eosin (HE) stained sections illustrate significant necrosis and deeply infiltrative characteristics (**Figures 2A, B**). The tumor penetrated the serosa with documented lymphovascular and perineural invasion. Metastases were identified in 9 of 20 resected lymph nodes, alongside multiple tumor deposits. The final pathological stage was pT4bN2bM0, Stage IIIC (AJCC 8th Edition). Immunohistochemistry (IHC) demonstrated loss of MLH1 and PMS2 proteins with retained MSH2 and MSH6 expression (**Figures 2C–F**), confirming dMMR status. Next-generation sequencing (NGS) of tumor tissue identified KRAS wild-type status, BRAF V600E mutation, and MSI-H status. Due to advanced age and personal preference, the patient initially declined adjuvant chemotherapy.

In April 2023, four months post−surgery, the patient presented with right lower quadrant pain. Abdominal CT revealed a soft tissue mass in the right lower quadrant with multiple retroperitoneal lymphadenopathies and invasion of the right ureter causing hydronephrosis (**Figure 1B**). A ureteral stent was placed on April 13, 2023. By April 25, 2023, symptoms worsened, and emergency CT showed rapid progression of the mass causing incomplete intestinal obstruction (**Figure 1C**). The diagnosis was revised to postoperative recurrence of ascending colon cancer (rT4bN2M1, Stage IV).

Given the patient’s frailty (cachexia, weight 44 kg) and the aggressive nature of the recurrence, conventional cytotoxic chemotherapy was deemed poorly tolerable. Based on the molecular profile (MSI−H, BRAF V600E), a combination of immunotherapy and targeted therapy was initiated on April 24, 2023. The regimen consisted of pembrolizumab (200 mg IV every 3 weeks) combined with dabrafenib (150 mg PO BID) and trametinib (2 mg PO QD).

Shortly after initiating therapy, the patient developed high−grade fever (up to 40 °C). A comprehensive workup including blood culture, C−reactive protein (CRP), serum amyloid A (SAA) excluded infectious etiologies; corticosteroids were not administered. The fever was attributed to the BRAF/MEK inhibitors (drug fever). Despite dose reduction and antipyretic support (ibuprofen), intermittent pyrexia persisted. Consequently, dabrafenib and trametinib were permanently discontinued on June 30, 2023. The patient continued with pembrolizumab monotherapy for maintenance.

**Figure 3:**
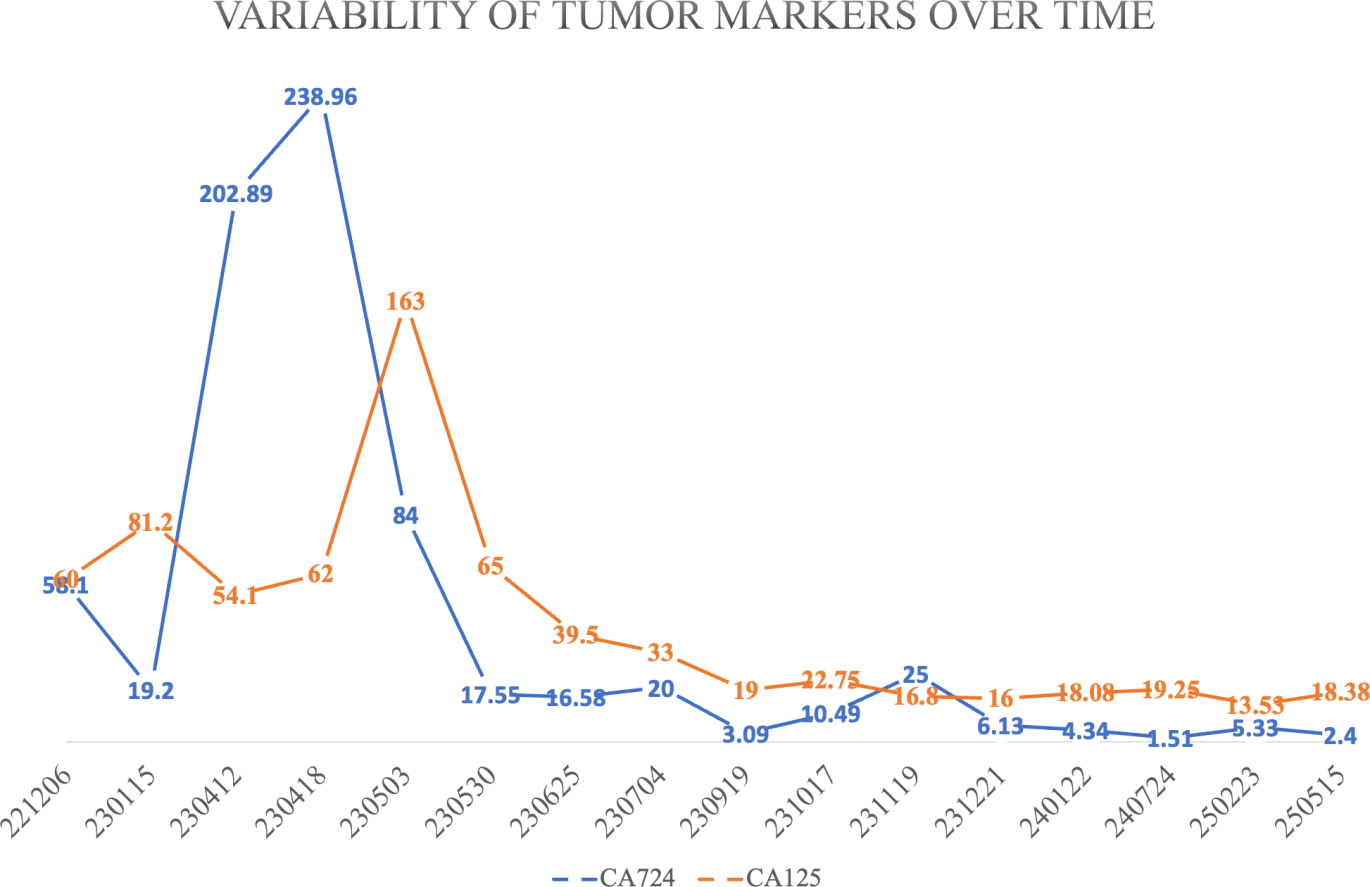
Kinetics of serum tumor markers. Graph illustrates the rapid decline in CA72−4 and CA125 levels following the initiation of combination therapy on April 24, 2023. Note the sharp drop in CA72−4 by mid−June 2023, coinciding with the period of targeted therapy administration.

**Figure 4:**
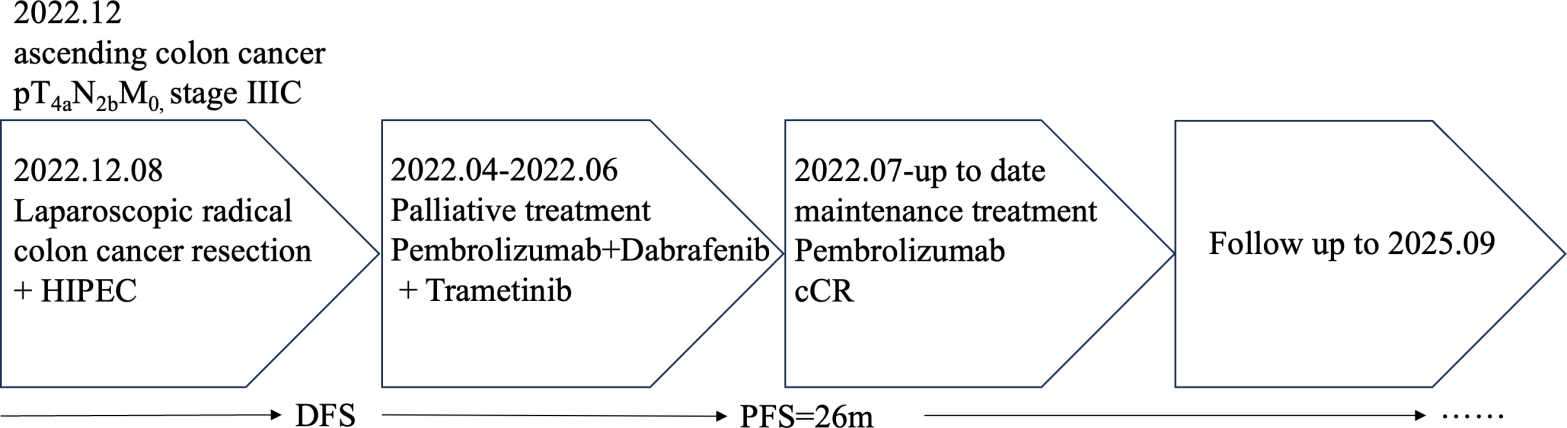
Summary of clinical timeline. Diagram depicts key clinical events: primary surgery (December 2022), disease recurrence (April 2023), initiation of triplet therapy (April 24, 2023), discontinuation of dabrafenib/trametinib (June 30, 2023) due to toxicity, and ongoing maintenance with pembrolizumab leading to a progression-free survival (PFS) of >26 months.

Tumor markers declined rapidly following the initiation of triplet therapy (**Figure 3**). Serial CT scans demonstrated continuous tumor shrinkage (**Figure 1D, E**). A radiographic complete response (CR) according to RECIST 1.1 criteria was achieved and confirmed by multidisciplinary review. As of the latest follow−up on September 9, 2025, the patient remains asymptomatic with no evidence of disease recurrence (**Figure 1F**), achieving a progression−free survival (PFS) of over 26 months(**Figure 4**).

## Discussion

CRC harboring concurrent MSI−H/dMMR and BRAF V600E represents a distinctive clinicomolecular subgroup enriched in right−sided primaries, older age, female sex, and aggressive histopathologic features, and is historically associated with inferior outcomes under conventional cytotoxic strategies (5–7). In our case, the rapid postoperative relapse and bulky locoregional/retroperitoneal progression were clinically concordant with the adverse biology suggested by the pathologic profile (poor differentiation, high proliferative index, lymphovascular/perineural invasion, nodal involvement). At the same time, the tumor’s MSI−H/dMMR status provided a strong biologic premise for ICIs, given the hypermutated phenotype, increased neoantigen burden, and T−cell–inflamed microenvironment typical of dMMR CRC (1, 10).

A key interpretive element in our patient is the dMMR immunophenotype (MLH1/PMS2 loss with retained MSH2/MSH6) together with BRAF V600E, which most often supports a sporadic MSI−H pathway (commonly linked to MLH1 promoter hypermethylation) rather than Lynch syndrome, and is frequently observed in right−sided tumors (6, 11, 12). This molecular context is clinically relevant not only for hereditary counseling, but also for therapeutic planning: MSI−H/dMMR predicts benefit from PD−1 blockade according to KEYNOTE−177 (2, 3), whereas BRAF V600E confers MAPK−driven aggressiveness and can shape an immunosuppressive milieu that may limit the durability of ICIs monotherapy in a subset of patients (7).

The biological rationale for combining immunotherapy with BRAF−directed therapy is supported by converging translational and clinical observations. Constitutive MAPK signaling downstream of BRAF V600E promotes tumor growth and may contribute to immune escape through multiple mechanisms, including impaired antigen presentation and recruitment/activation of immunosuppressive cell populations, such as MDSCs/Tregs, while MAPK pathway inhibition can enhance tumor antigenicity, increase intratumoral T−cell infiltration, and induce adaptive upregulation of checkpoint pathways such as PD−L1—thereby creating a context in which PD−1 blockade may be synergistic (8, 9). This immunomodulatory premise underpins ongoing efforts to combine PD−1 blockade with BRAF/EGFR−targeted regimens in MSI−H/BRAF−mutant mCRC, including the randomized phase II SEAMARK study comparing pembrolizumab alone versus pembrolizumab plus encorafenib/cetuximab as first−line therapy for MSI−H/dMMR, BRAF V600E mCRC (9). Although standard CRC biology differs from melanoma in the degree of EGFR feedback activation, the broader concept that targeted therapy can “recondition” the immune microenvironment provides a plausible explanation for deep and durable responses in selected patients when targeted and immune therapies are combined or sequenced strategically (8, 9).

Clinically, ICI monotherapy is an established first−line standard for MSI−H/dMMR mCRC (2, 3). However, real−world evidence suggests that BRAF V600E may influence response durability rather than initial response probability. In the largest international retrospective cohort to date (909 dMMR/MSI−H mCRC treated with ICIs), BRAF V600E was not independently associated with inferior PFS/OS across all treatment lines after multivariable adjustment; nevertheless, among patients receiving first−line ICI, BRAF V600E was associated with a significantly higher rate of secondary resistance (progression after 6 months), and survival beyond a 6−month landmark was shorter in BRAF−mutant tumors—an effect that appeared attenuated in patients treated with combined anti−PD−1/anti−CTLA−4 (7). These findings provide an informative framework for our case: the decision to intensify upfront therapy with PD−1 blockade plus MAPK−targeted therapy in a rapidly progressive, frail patient can be viewed as a biologically and clinically rational attempt to reduce early tumor burden while potentially lowering the risk of late escape suggested in BRAF−mutant MSI−H disease treated with PD−1 monotherapy alone (7, 9).

Our patient achieved a rapid and durable complete response despite discontinuation of dabrafenib/trametinib after a short exposure due to pyrexia. This observation raises the hypothesis that even brief MAPK pathway suppression may have served as an “immune−priming” event, facilitating sustained immune control under continued pembrolizumab maintenance—consistent with the concept that targeted therapy can modulate antigen presentation and T−cell trafficking, thereby potentiating subsequent or concurrent PD−1 blockade (8, 9). While causality cannot be established from a single case, our clinical course is directionally consistent with emerging interest in combination and sequencing approaches in this subgroup. Complementary case−level evidence also supports meaningful ICI activity in MSI−H/BRAF V600E CRC even in rare aggressive histologies: a recent report of MSI−H hepatoid adenocarcinoma with BRAF V600E demonstrated prolonged disease−free survival with adjuvant PD−1 blockade and minimal immune−related toxicity (13).

When comparing alternative therapeutic approaches, cytotoxic chemotherapy is frequently poorly tolerated in very elderly or frail patients and is often less effective in BRAF V600E disease; moreover, MSI−H tumors can exhibit limited benefit from fluoropyrimidine−based strategies in some settings (6, 14). BRAF−targeted regimens (classically BRAF plus EGFR inhibition, with or without MEK inhibition) have demonstrated activity in previously treated BRAF V600E mCRC (4, 12), but their durability may be limited relative to successful ICI in MSI−H disease. Accordingly, contemporary clinical development is increasingly focused on integrating targeted therapy with ICI earlier in the disease course to combine rapid cytoreduction with durable immune control (9), a strategy that aligns with the clinical objectives in our patient given her tumor burden, symptom trajectory, and chemotherapy ineligibility.

Finally, toxicity management and real−world clinical decision−making are central to the interpretability of combination strategies. Pyrexia is a well−recognized adverse event of BRAF/MEK inhibition and, in frail patients, may necessitate prompt evaluation (including exclusion of infection), supportive care, dose interruption/reduction, and, when persistent, discontinuation—while continuing the most tolerable and mechanistically essential therapy (e.g., PD−1 blockade in MSI−H disease) (4, 9). Our case underscores a pragmatic treatment paradigm: early intensification to achieve disease control in a high−risk molecular subtype, followed by de−escalation to maintain efficacy with acceptable tolerability. In the context of evidence that BRAF−mutant MSI−H mCRC may experience late escape on PD−1 monotherapy (7), this approach may be particularly relevant for patients with aggressive clinicopathologic features, high tumor burden, or rapid disease kinetics—although prospective trials such as SEAMARK are needed to define optimal sequencing, duration, and patient selection (9).

## Conclusion

This case illustrates the transformative potential of precision medicine in molecularly defined CRC subtypes. For patients with MSI-H and BRAF V600E co-mutated mCRC, combined BRAF/MEK inhibition and immunotherapy represents a promising first-line strategy capable of reversing innate immunosuppression and inducing durable remissions. Importantly, our findings suggest that induction with targeted therapy followed by ICI maintenance is a viable strategy for patients who cannot tolerate prolonged combination treatment.

## Data Availability

The raw data supporting the conclusions of this article will be made available by the authors, without undue reservation.
